# Notes and News

**Published:** 1887-05-21

**Authors:** 


### Notes and News.

COLLECTED AND COMMUNICATED.

THE festival dinner on behalf of the Middlesex Hospital (fixed for Wednesday last) has been unavoidably postponed.

THE Queen has not only consented to become patron of the French Hospital, but has sent a handsome donation towards the building fund of the new premises of this admirable institution, to be erected in Shaftesbury Avenue.

THE Banner Sanitation Company have taken over the agency for Doecker's Patent Portable Hospitals and Huts, a description of which will be found in our advertising columns.

THE Islington and North-East London Local Committee of the Hospital Saturday Fund are appealing for the assistance of fifty ladies who will act as collectors for that neighbourhood on Hospital Saturday, June 11th. Mr. J. E. Crewe, the hon. sec., 334, Caledonian Road, N., will gladly hear from any ladies willing to render this help.

THE Committee of the Great Northern Central Hospital (which is now in course of erection in the Holloway Road) have received from the Committee of the Portman Cinderella Dances a cheque for £150 in aid of the building fund, the proceeds of these dances during the past season. To commemorate the gift, one of the beds in the new hospital will be called the "Portman Cinderellas."

IN aid of the Morley Convalescent Home for Working Men at St. Margaret's, Dover, an excellent entertainment was given at the new Chelsea Town Hall, on Thursday, 19th inst., under the patronage of the Duchess of Albany, the Prince and Princess Christian, Prince and Princess Henry of Battenberg, the Princess Frederica of Hanover and Baron von Pawel-Rammingen, the Duke of Cambridge, and the Duke and Duchess of Teck. On 27th inst., by the kind permission of the Duke of Westminster, another entertainment will be given at Grosvenor House.

THE fifth annual demonstration of the Temperance Trades and Friendly Societies in aid of the Victoria Hospital for Children has been fixed to take place early in July. The secretary, Mr. William Kimber, of 40, Markham Square, S.W., will be pleased to hear from any Societies wishful to take part in his laudable movement.

THE Islington Jubilee Committee have resolved to endeavour to raise by voluntary subscriptions £20,000, to be devoted to the benefit of the Great Northern Central Hospital, now in course of erection. Sir James Tyler, Mr. John Blount Price, and Mr. R. A. Newbon have been appointed joint honorary treasurers, and Mr. W. F. Dewey honorary secretary to the Fund. There is very pressing need for the new hospital. Last month twenty-seven cases had to be rejected simply through want of accommodation.

THE Committee of the Great Northern Central Hospital in the Caledonian Road have received a donation of one thousand guineas towards their building fund from Mr. R. A. Newbon. The generous donor, who is an inhabitant of Islington, wishes his gift to be applied to the endowment of a bed in the new hospital in the Holloway Road, in memory of his sister, lately deceased. We believe that the sum of £35,000 is still required to complete these buildings, and we trust Mr. Newbon's good example may yet be followed by many other wealthy persons.

SIR EDWARD CECIL GUINNESS has contributed the sum of five thousand pounds towards the scheme now being promoted in Dublin for the erection of a Consumption Hospital in that city. The Irish climate is peculiarly liable to foster this disease, and the proposal is one worthy of all support. If it be a success, as so prosperous a start seems to promise that it will be, we hope its management will be placed upon a thoroughly sound basis from the commencement, and that it may give a pattern to, and not follow the example of, most Irish hospitals in this all-important particular.

WE are very pleased to learn that the proposal to build, by means of funds contributed by children, a new wing to complete the Children's Hospital in Great Ormond Street, as the Children's Jubilee Offering to the Queen (the patron of the hospital), is meeting with much success. The Jews' free school at Spitalfields, perhaps one of the very poorest quarters of London, has sent a contribution of £4 11*s.* 7*d.*, and sums have been received from a large number of other schools, including Eton. English children resident abroad and in the colonies have also helped. One boy at a public school, having gained a scholarship, has sent £5 as a thank-offering. The patronesses of the fund include the Princess of Wales, Princess Mary, Duchess of Teck, and the Princess Victoria of Teck. The Rev. T. Teignmouth Shore, 31, Montpelier Square, S.W., is the treasurer to the fund. Miss Henderson, 4, Gledhow Gardens, South Kensington, is the hon. sec.

The following vacancies are announced this week, the amount of the salary being placed in each case after the name of the institution :—

**Resident Medical Officers.**

Liverpool Northern Hospital. Assistant House Surgeon. Doubly-qualified.—£70.
Newcastle Infirmary. House Physician. Doubly-qualified. —£100.
Rotherham Hospital. Assistant. No salary.
West London Hospital. House Physician and two House Surgeons. Registered.

**Non-resident Medical Officers.**

Birmingham Eye Hospital. Dispenser.—£70.
Evelina Hospital, S.E. Registrar and Cloroformist. Qualified. —£30.
Staines Union. Medical Officer of Health. Qualified.—£75.
Victoria Hospital, Chelsea. Assistant Surgeon.

**Matrons, etc.**

Chelsea Workhouse Infirmary.—£80.
Clinical Hospital, Park Place, Manchester.—£35.
Pendlebury Hospital, Manchester.—£100.

**Trained Nurses.**

Beckett Hospital, Barnsley. One.—£25.
Cancer and Skin Hospital, Liverpool. One.
Devon and Exeter Hospital. Two.—£25 each.
Exeter Hospital. Two.—£25.
Fever Hospital, Middlesborough. Charge Nurse.—£30.
Fitzroy House Hospital. One day, one night.
Kidderminster Infirmary. One night.—£20.
Leeds Nurses' Home. One.—£30.
Leicester Fever Hospital. Charge Nurse.—£25.
Monmouth Hospital. One.—£25.
Seacombe Cottage Hospital, Birkenhead. One.

**Probationer.**

Newark Hospital, Notts. One.

---

A very pretty bazaar was held last week at King's College in aid of the hospital in connection with this institution. Though the hospital is happily now free from debt, the recent structural changes have caused an extraordinary expenditure of £6,000, and it was to assist in raising this sum that the bazaar was arranged. The Princess Louise, accompanied by the Marquis of Lorne and Lady Mary Glyn, attended and declared the bazaar open. There was a large attendance, and it is to be hoped that a substantial sum has been raised. An interesting incident occurred just after the opening proceedings. A little boy, of about four years of age, who has recently had one of his legs amputated, presented a beautiful bouquet to the Princess, who smilingly accepted the gift.

---

In presenting the 151st annual report, the committee of the Bristol Royal Infirmary call attention to the fact that the expenditure for 1886 exceeded the income by £1,680. Of this large deficit, however, £880 was an exceptional outlay—for drainage, improvements in the operation theatre, etc.—so that the excess of ordinary expenditure over income was about £800. The income for the year, exclusive of a legacy of £5,000, was £10,771, and the expenditure £11,600, or about £374 less than in 1885. Readers will learn with surprise that for the last thirty-six years, in spite of the large increase which has taken place amongst the population of both Bristol and Clifton, the subscriptions to the infirmary have shown no material alteration. Who is to blame for this remarkable state of things, we wonder? The total number of patients admitted in 1886 was 32,419, of whom 3,518 were in-patients. It is stated that very extensive painting and repair will have to be done to the interior of the building during the current year, and the committee appeal to the public to carry them through this necessary work without further reduction of their invested funds.

The Building Committee for the new Provident Dispensary at Bedford have accepted the tender of Mr. J. P. White, amounting to £1,626. Nearly the whole of the sum required for the new building, including the furniture, has now been raised.

---

Last Saturday was Hospital Saturday in Birmingham. Collections were very extensively made in the various workshops in aid of the local hospitals. Last year Hospital Saturday in Birmingham yielded £6,703, and it is anticipated that this year that figure will be exceeded.

---

A private subscription ball has been arranged to take place at Prince's Hall, Piccadilly, on 1st June, the proceeds of which are to be devoted to Mrs. Black's Cottage Hospital. Tickets may be obtained from the hon. sec., 5, Hazlitt Road, West Kensington Park.

---

The Lord Mayor has sent a cheque for ten guineas to the Morley House Seaside Convalescent Home for Working Men, St. Margaret's, Dover. His lordship has also consented to become a patron of the Banner Service which is to be held next Sunday at the Pavilion Theatre in aid of this institution.

---

The Cork Women's and Children's Hospital is, we believe, one of the most efficiently managed institutions in Ireland. The annual report for 1886 is certainly one of an instructive character. There were 308 in-patients treated last year, the average cost per bed being £31 4*s.* 8*d.*, including not only the cost of maintaining the patient, but also a share of the general expenses of the hospital. A very great improvement has been carried out at the hospital by the introduction of a separate ward system for Catholic and Protestant patients.

---

For the fifth successive year the various members of Friendly and Trade Societies of the north-western district held their "demonstration" in aid of their two local hospitals—University College and the North-West London. The different orders, such as the Buffaloes, Druids, Foresters, and Shepherds, appeared in their regalia, creating a very picturesque appearance. The procession started from the "Bull and Gate," Kentish Town, and marched to St. Pancras Church, where the service was held. Dr. Spence, Dean of Gloucester, preached. During the past four years the Friendly Societies of this district have collected for the two hospitals the handsome sum of £869.

---

It has been decided to enlarge the Boston Cottage Hospital by the addition of a wing at the west-end. The little hospital was originally built and furnished for twelve beds, but the pressure of cases has led the committee to squeeze in six more, which has meant, of course, a certain amount of overcrowding. The need for making a permanent enlargement has been felt for some time past, and uninvited contributions and promises of help have been coming in, one lady offering to furnish one of the new wards at her own expense. The new wing will, it is estimated, cost between £600 and £700.

The following sums have been recently received by the various Medical Charities, the name of the donor or the source from which they are obtained being given in each instance after the name of the Institution :—

**Donations.**

British Home for Incurables, Clapham. Company of Merchant Taylors, £10 10*s*.
East London Hospital for Children. Company of Merchant Taylors, £21.
Lock Hospital, Harrow Road. A. M., £10.
National Hospital for Consumption, Ventnor. Mr. C. J. Bunvon, £21.
North-Eastern Hospital for Children. Company of Merchant Taylors, £10 10*s*. Vestry of St. Edmund the King and Martyr, £10 10*s*.
North London Consumption Hospital. Rt. Hon. Sir Henry Holland, M.P., £10.
North London Hospital, Gower Street. J. B. (per Dr. Irving Menzies), £100. Mr. Wm. McFarlane, £10 10*s*. Mr. R. Kaye Gray, £25. Mr. T. F. Gibson, £20. Mrs. Nathaniel Montefiore, for further endowment of the Leonard and Montefiore Cot, £200; for general purposes of the hospital, £100. "People's Contribution Fund," £105. Mr. H. Head, £10 10*s*. Mr. J. W. Green, £10 10*s*. Mr. M. H. Gray, £10 10*s*.
Seamen's Hospital, Greenwich. Clothworkers' Company, £20.
West London Hospital, Hammersmith. Employés of the Brentford Gas Co., £15 2*s*. 1*d*.

**Legacies.**

British Home for Incurables, Clapham. Miss Winkle, £568 10*s*.

---

Yesterday (Friday) a Festival Dinner was held at the Hôtel Métropole, in commemoration of the twentieth anniversary of the foundation of the Victoria Hospital for Children, under the presidency of the Duke of Cambridge. The committee are very anxious to raise a sum of £4,000, the amount of the debt outstanding on the new Out-Patients' Wing, which the Prince and Princess of Wales opened last June. The total cost of this valuable addition to the hospital (including the furnishing) has amounted to about £8,000.

---

Of all sad sights, perhaps the interior of a hospital for incurable cases of disease is the most distressing. In the Royal Hospital for Incurables at West Hill, Putney, there is at the present moment an old woman, seventy-four years of age, who for exactly half a century has been a confirmed invalid, the victim of rheumatism, induced by a chill. But such a case as this is by no means one of the worst. At the annual dinner, held at the Albion Tavern the other evening, in aid of this institution, Sir Edward Clarke remarked that, in going over the wards with Mr. Allcroft, the conviction was borne to his mind that this was one of the grandest works which human charity ever originated. The helpless men and women there are, many of them, great sufferers; but though in pain, they are happy. Sir Edward said he did not observe a single unhappy face, an experience which we can ourselves corroborate. Besides the 200 patients at West Hill, there are scattered through the country some 500 others, pensioners in the receipt of £20 a year. This pecuniary assistance enables the sufferers to command some care and comfort to ease their cheerless lot. The seaside branch at St. Leonards, which has been generously supported by Mr. Allcroft for the whole of a year, is to become a permanent feature of the hospital, and for this purpose a sum of £3,000 is wanted. This year—in commemoration of the Jubilee—the number of candidates to be admitted will be increased from twenty-five to forty.

The admirable generosity of Mr. J. S. Morgan, already chronicled in our columns, will, we trust, produce many imitations all over the country. One such is to be found in the person of Mr. John Geddes, a prominent member of the local Liberal party in Southport, who has offered to give £1,000 to start a fund for building a new infirmary. The question of erecting a new hospital has frequently been mooted, but hitherto nothing tangible has resulted. It is, however, hoped and believed that Mr. Geddes's example will lead to the speedy completion of this useful project.

---

Police-Sergeant Barker, who was so cruelly used by the Finchley burglars, has made a rapid recovery at the Royal Free Hospital, whence he has been removed to the Merchant Taylors' Convalescent Home at Bognor, to completely rehabilitate his strength. Messrs. C. Wright and Sons, of 108, New Bond Street, the well-known surgical instrument makers, have promised to give the unfortunate officer one of their best artificial limbs. Barker's conspicuous bravery has been readily acknowledged by the benevolent public, for Mr. J. S. Blyth, the Secretary to the Royal Free Hospital, states that the subscriptions he has received (including a gratuity of £50 awarded him by the Home Office) amount to £286 11*s*. 1*d*. Dr. James Turle (whose house was the object of attack) adds that the North Finchley Testimonial Fund now amounts to £222 17*s*. 6*d*.

---

Dr. Robertson presided at the annual meeting of supporters of the Devonshire Hospital, Buxton, on Saturday, May 7th, and in the course of his address pointed out the great usefulness of the institution, which gave the possibility of restoring health to two thousand rheumatic patients year by year. From May 1st, 1886, to April 30th, 1887, 2,459 in-patients were admitted, of whom no less than 2,238 had been sent away as improved, whilst 121 remained still on the books at the end of the period. After some discussion the meeting adjourned in order to inspect the new massage baths, which have been constructed at the expense of the Duke of Devonshire for the benefit of poor patients. These baths are situated in the colonnade of the hospital, have just been completed, and are likely to prove of inestimable value to many poor invalids.

---

Entertainments in aid of the funds of the Eastern Counties Children's Convalescent Home were given on the evenings of the 9th and 10th of May at the Royal Aquarium, Yarmouth, and there was a large attendance upon each occasion. The audience was also augmented by the occupants of a special train from Norwich, which had been provided. On the first night *Pygmalion and Galatea*, and *A Husband in Clover*, were performed, and on the second night the audience was gratified by a very able representation of *Caste*. We trust that the ladies and gentlemen who kindly consented to give their services in so good a cause were gratified by being able to present the treasurer of the Home with a substantial sum as the result of their exertions.

